# A new cryptic species of *Anolis* lizard from northwestern South America (Iguanidae, Dactyloinae)

**DOI:** 10.3897/zookeys.794.26936

**Published:** 2018-11-01

**Authors:** Mario H. Yánez-Muñoz, Carolina Reyes-Puig, Juan Pablo Reyes-Puig, Julián A. Velasco, Fernando Ayala-Varela, Omar Torres-Carvajal

**Affiliations:** 1 Unidad de Investigación, Instituto Nacional de Biodiversidad. Rumipamba 341 y Av. de los Shyris. Casilla postal: 17-07-8976. Quito, Ecuador Instituto Nacional de Biodiversidad Quito Ecuador; 2 Instituto de Zoología Terrestre, Museo de Zoología, Instituto BIOSFERA, Colegio de Ciencias Biológicas y Ambientales COCIBA, Universidad San Francisco de Quito USFQ, Diego de Robles y Vía Interoceánica, 170901, Quito, Ecuador Universidad San Francisco de Quito Quito Ecuador; 3 Fundación Red de Protección de Bosques ECOMINGA, Fundación Oscar Efrén Reyes, Departamento de Ambiente, Calle 12 de Noviembre N° 270 y Calle A. Martínez, Baños, Ecuador Fundación Red de Protección de Bosques ECOMINGA Baños Ecuador; 4 Museo de Zoología “Alfonso L. Herrera”, Facultad de Ciencias, Universidad Nacional Autónoma de México, Mexico city, Mexico Universidad Nacional Autónoma de México Mexico Mexico; 5 Museo de Zoología, Escuela de Ciencias Biológicas, Pontificia Universidad Católica del Ecuador, Avenida 12 de Octubre 1076 y Roca, Quito, Ecuador Pontificia Universidad Católica del Ecuador Quito Ecuador

**Keywords:** *Anolisdracula* sp. n., diversity, morphology, phylogeny, Squamata, taxonomy, *Anolisdracula* sp. n., diversidad, filogenia, morfología, Squamata, taxonomía

## Abstract

A new species of *Anolis* lizard from the Andean slopes of southwestern Colombia and northwestern Ecuador, from between 1187 and 2353 m in elevation, is described. The new species can be distinguished from other *Anolis* in squamation, cranial osteology, hemipenial morphology, and nuclear and mitochondrial DNA. The new species is sister to *Anolisaequatorialis*, and it is suggested that previous records of *A.aequatorialis* in Colombia correspond to the new species described herein.

## Abstract (es)

Describimos una nueva especie de *Anolis* de las estribaciones de los Andes del suroccidente de Colombia y noroccidente de Ecuador, entre los 1187 y 2353 metros de elevación. La nueva especie puede ser distinguida de otros *Anolis* por caracteres de escamación, osteología craneal, morfología hemipenial, y ADN nuclear y mitocondrial. La nueva especie es hermana de *A.aequatorialis*, por lo que sugerimos que los registros previos de *A.aequatorialis* en Colombia corresponden a la nueva especie descrita en este artículo.

## Introduction

*Anolis* lizards (anoles) are members of a diverse clade with 427 recognized species (Losos and Ricklefs 2009, Poe et al. 2017, Uetz and Hošek 2018). The phylogenetic relationships among species of anoles have been controversial for many decades (Guyer and Savage 1986, Cannatella and de Queiroz 1989, Guyer and Savage 1992, Nicholson 2002, Poe 2004, Nicholson et al. 2012) and a phylogenetic hypothesis based on morphology and DNA sequence data including most species of *Anolis* was not available until recently (Poe et al. 2017).

The taxonomic knowledge on Ecuadorian and Colombian anole lizards increased significantly in the 60’s and 70’s through contributions by Ernest Williams and other herpetologists, who described almost 70% of all anole species (Williams 1965a, b, 1966, 1967, 1974, 1975, 1979). Notably, the rate of anole species discovery increased again in the last decade (Ugueto et al. 2007, Köhler et al. 2007, Köhler 2010, Torres-Carvajal et al. 2010, 2018, Ayala-Varela and Torres-Carvajal 2010, Ayala-Varela and Velasco 2010, Grisales-Martinez et al. 2017). Most recent descriptions are based on morphological and molecular evidence (Ayala-Varela et al. 2014; Guarnizo et al. 2016; Grisales-Martinez et al. 2017). However, comparative analyses of osteology and hemipenial morphology are usually not presented, with a few exceptions (Köhler et al. 2014; McCranie and Köhler 2015).

Herein we describe a new cryptic species of *Anolis* from northern Ecuador and southern Colombia, similar in morphology to *A.aequatorialis* Werner, 1894. In addition, we use DNA sequence data to infer its phylogenetic position within the *Dactyloa* clade (Poe et al. 2017).

## Materials and methods

### Taxon sampling

We examined specimens of *Anolis* within the *Dactyloa* clade (Poe et al. 2017) from Colombia and Ecuador, housed in Ecuador: the División de Herpetología del Instituto Nacional de Biodiversidad (DHMECN), Quito; the Museo de Zoología de la Pontificia Universidad Católica del Ecuador (QCAZ), Quito and in Colombia: the Colección Herpetológica de la Universidad del Valle del Cauca (UVC), Cali; the Museo de Herpetología de la Universidad de Antioquia (MHUA), Antioquia; the Colección de Reptiles, Instituto de Ciencias Naturales (ICN), Bogota; and the Colección de Herpetología del Instituto Alexander von Humboldt (IAvH), Villa de Leyva. All specimens examined are listed in Appendix 1, and we mapped all records using ArcMap 10.5.1 (ESRI, Inc.) with a WGS84 datum and with Universal Transverse Mercator conformal projection.

We adopted the unified species concept (de Queiroz 2007), which is operationalized based on substantial and consistent differences between populations, and followed Wiens and Servedio (2000) in recognizing populations as distinct evolutionary lineages, based on the frequency of traits allowing polymorphism. In addition, we considered monophyly as a strong evidence for recognizing populations as new species.

### Morphological data

We used the character terminology proposed by Williams et al. (1995) for scale characters and measurements. Specimens were sacrificed by spraying benzocaine directly in the mouth, fixed in 10% formalin, and preserved in 70% ethanol. Tissue samples (liver, muscle tissue, or scales) were extracted before fixing and placed in Eppendorf tubes with 96% ethanol. Sex determination was based on the presence of hemipenes, dewlap size and gonad inspection. Hemipenes were extracted from recently collected adult males, or everted from fixed specimens, using the method described by Pesantes (1994), with the modifications proposed by Betancourt et al. 2018. In the majority of specimens, the left organ was removed with a subcaudal incision, and submerged in sodium dodecyl sulfate for 24 hours. Skulls were prepared using dermestid beetles for two days until the bones were free of muscle tissue, and then degreased with sodium dodecyl sulfate for 24 hours. Data of color in life were obtained from field notes and photographs. All measurements were taken with a digital caliper with a precision of ±0.01 mm.

### Stomach contents

From the DHMECN material (n = 18) we removed the stomach contents with a ventrolateral incision to the stomach of the preserved specimens. The stomach contents of each anole were placed in a 4% formaldehyde solution in eppendorf tubes. The identification of samples was accomplished through a stereomicroscope. We determined the stomach content at the order and family level.

### DNA sequence data

Total genomic DNA was digested and extracted from liver or muscle tissue using a guanidinium isothiocyanate extraction protocol. Tissue samples were first mixed with Proteinase K and a lysis buffer and digested overnight prior to extraction. DNA samples were quantified using a Nanodrop ND-1000 (NanoDrop Technologies, Inc.), re-suspended and diluted to 25 ng/ul in ddH_2_O prior to amplification.

Using primers and amplification protocols from the literature (Folmer et al. 1994, Kumazawa and Nishida 1993, Macey et al. 1997, Schulte and Cartwright 2009), we obtained 2807 nucleotides (nt) representing the nuclear gene recombination-activating gene 1 (RAG1, 811 nt), as well as the mitochondrial genes Cytochrome c oxidase I (CO1, 655 nt) and a continuous fragment including NADH dehydrogenase subunit 2 (ND2, 1038 nt), tRNA^Trp^, tRNA^Ala^, tRNA^Asn^, tRNA^Cys^ (282 nt), and the origin of the light-strand replication (OL, 29 nt). New sequence data were obtained from three individuals of the new species and added to the dataset used by Torres-Carvajal et al. (2018). Gene regions of taxa sequenced in this study along with their GenBank accession numbers are shown in Table 1. Information on other taxa included in the phylogenetic analyses is available in Torres-Carvajal et al. (2018).

**Table 1.** *Anolisdracula* sp. n. vouchers, locality data, and GenBank accession numbers for gene sequences generated for this study.

Paratype voucher	GenBank accession number		
	COI	ND2	RAG1
QCAZ 4387: Ecuador: Carchi: San Pablo river, close to Chical. 0°54'10.87"N, 78°9'46.22"W, 1399 m.	MH727638	MH733476	MH727641
QCAZ 4405: Ecuador: Carchi: Maldonado, Sendero Ecológico Teldibi. 0°54'46.83"N, 78°6'28.15"W, 1477 m.	MH727637	MH733475	MH727640
QCAZ 4411: Ecuador: Carchi: Maldonado, Sendero Ecológico Teldibi. 0°54'46.83"N, 78°6'28.15"W, 1477 m.	MH727636	MH733474	MH727639

### Statistical analyses

Given that the new species is very similar to *Anolisaequatorialis*, we performed a comparison using univariate t-tests for independent samples to evaluate quantitative differences between the two species (for normal data), and a Wilcoxon-Mann Whitney test for differences in squamation (for non-normal data). We conducted the Shapiro-Wilk normality test for the distribution of the data (Table 2). Statistical analyses were conducted in R (R Core Team 2016).

**Table 2.** Shapiro-Wilk normality test for measurements and lepidotic characters of *Anolisdracula* sp. n. Asterisks indicate the degree of significance, * α = p < 0.05, ** α = p < 0.01.

Character	Shapiro-Wilk normality test *p* (α 0.05)	
	*A.dracula* sp. n.	* A. aequatorialis *
Scales between second canthals	0.00423**	3.83E-05**
Postrostrals	0.0002318**	0.02137*
Loreal rows	2.42E-07**	3.60E-08**
Scales between supraorbital semicircles	0.0001281**	3.60E-05**
Scales between interparietal and semicircles	2.12E-06**	7.18E06**
Supralabials to below center of eye	9.79E-08**	4.39E-08**
Postmentals	6.88E-06**	0.006722**
Lamellae under phalanges III-IV of fourth toe	0.02271*	0.001285**
Head length	0.4423	0.4422
Head width	0.364	0.1512
Head height	0.2718	0.8316
Jaw length	0.6772	0.5041
Snout length	0.947	0.2273
Forelimb length	0.918	0.9827
Hindlimb length	0.944	0.873
Axilla-groin length	0.126	0.07
Snout-vent length	0.1187	0.5671
Tail length	0.7504	0.07
Dewlap length	0.2672	0.0743
Dewlap height	0.6148	0.06
Interparietal scale length	0.729	0.4702
Tympanum length	1.647	0.8122

### Phylogenetic analyses

Editing, assembly, and alignment of sequences were performed in Geneious ProTM 5.3 (Drummond et al. 2010). Genes were combined into a single dataset with eleven partitions, three per protein coding gene corresponding to each codon position, one with all tRNAs, and one with the OL. The best partition strategy along with the corresponding models of evolution were obtained in PartitionFinder 1.1.1 (Lanfear et al. 2012) under the Bayesian information criterion.

Phylogenetic relationships were assessed under a Bayesian approach in MrBayes 3.2.0 (Ronquist and Huelsenbeck 2003). Four independent analyses were performed to reduce the chance of converging on a local optimum. Each analysis consisted of 20 million generations and four Markov chains with default heating values. Trees were sampled every 1000 generations resulting in 20,000 saved trees per analysis. Stationarity was confirmed by plotting the –ln L per generation in the program Tracer 1.6 (Rambaut et al. 2013). Additionally, the standard deviation of the partition frequencies and the potential scale reduction factor (Gelman and Rubin 1992) were used as convergence diagnostics for the posterior probabilities of bipartitions and branch lengths, respectively. Adequacy of mixing was assessed by examining the acceptance rates for the parameters in MrBayes and the effective sample sizes (ESS) in Tracer. After analyzing convergence and mixing, 2000 trees were discarded as “burn-in” from each run. We then confirmed that the four analyses reached stationarity at a similar likelihood score and that the topologies were similar, and used the resultant 72,000 trees to calculate posterior probabilities (PP) for each bipartition on a 50% majority rule consensus tree. We calculated ND2 uncorrected genetic distances in PAUP* 4.0 (Swofford 2002).

## Results

### Anolis dracula sp. n.

Taxon classificationAnimaliaSquamataIguanidae

http://zoobank.org/86C3BFDA-80D6-4A21-BD03-B6DC8B9FD1F9

**Figure 1.**
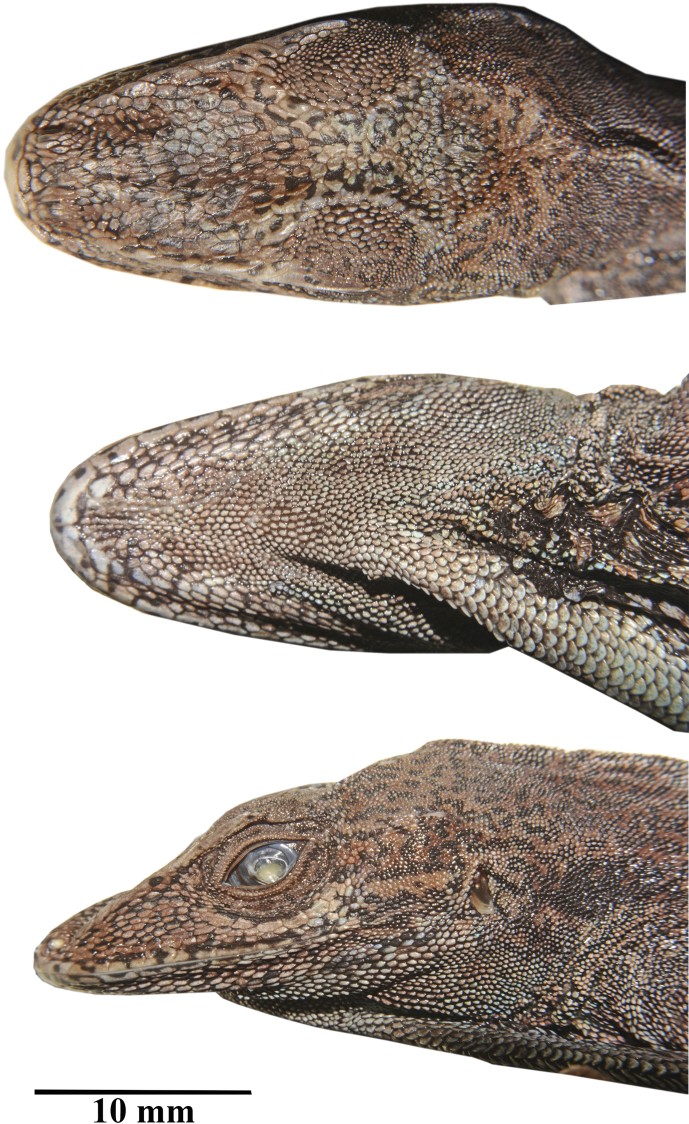
*Anolisdracula* sp. n. Holotype DHMECN 12579, 91 mm SVL. Dorsal (top), ventral (middle) and lateral (bottom) views of head. Photographs by Mario H Yánez-Muñoz.

**Figure 2.**
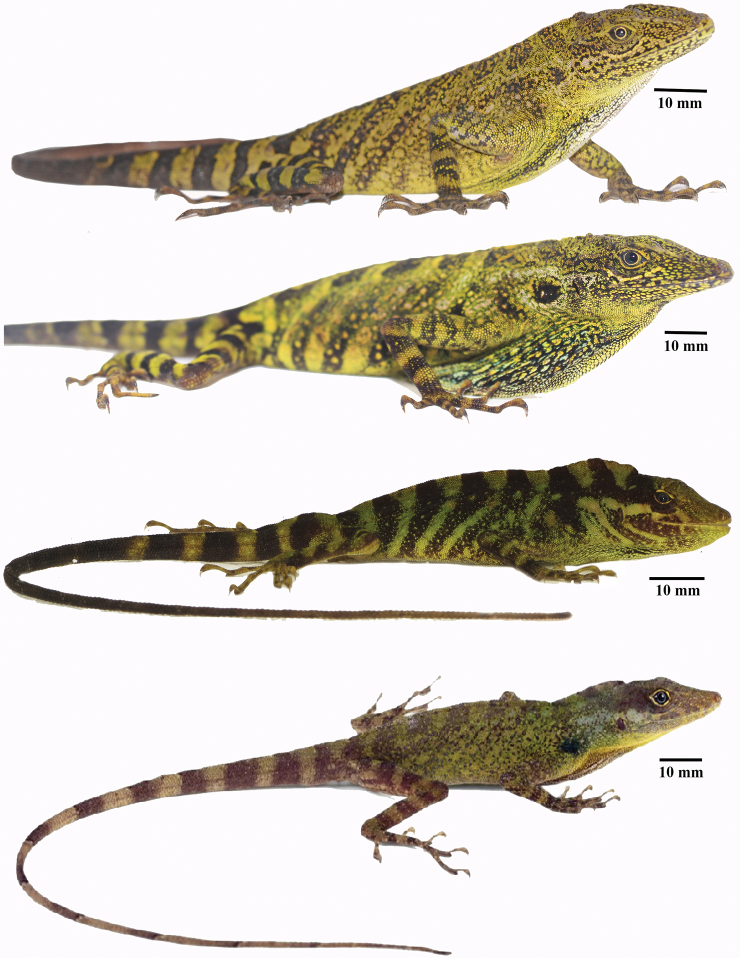
Comparison of *Anolisdracula* sp. n. with similar species. From top to bottom: male of *A.dracula*, Holotype DHMECN 12579, 91 mm SVL; male of *A.aequatorialis*, not collected; male of *A.fitchi*DHMECN 11628, 74 mm SVL, male of *A.podocarpus*QCAZ 10126, 87 mm SVL. Photographs, from top to bottom, by Mario Yánez-Muñoz, Carolina Reyes-Puig, Mario Yánez-Muñoz, and Fernando Ayala Varela.

**Figure 3.**
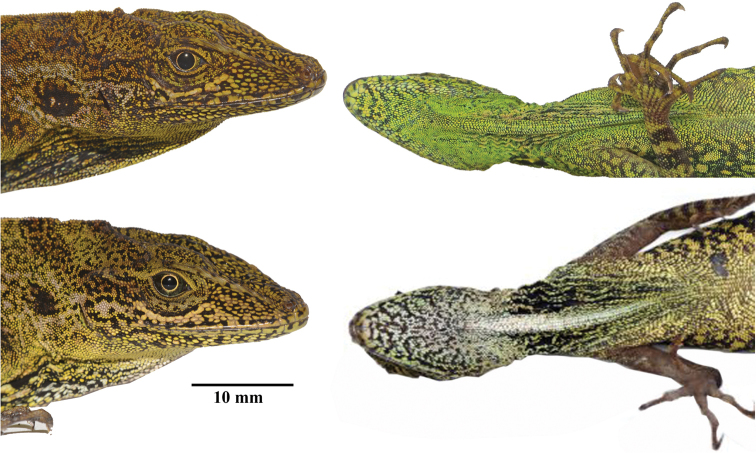
Head and throat region of *Anolisaequatorialis* (top, individual not collected) and *A.dracula* sp. n. (bottom left, DHMECN 12579, holotype, bottom right QCAZ 4365). Photographs by Mario Yánez Muñoz, Carolina Reyes-Puig, and Santiago Ron.

**Figure 4.**
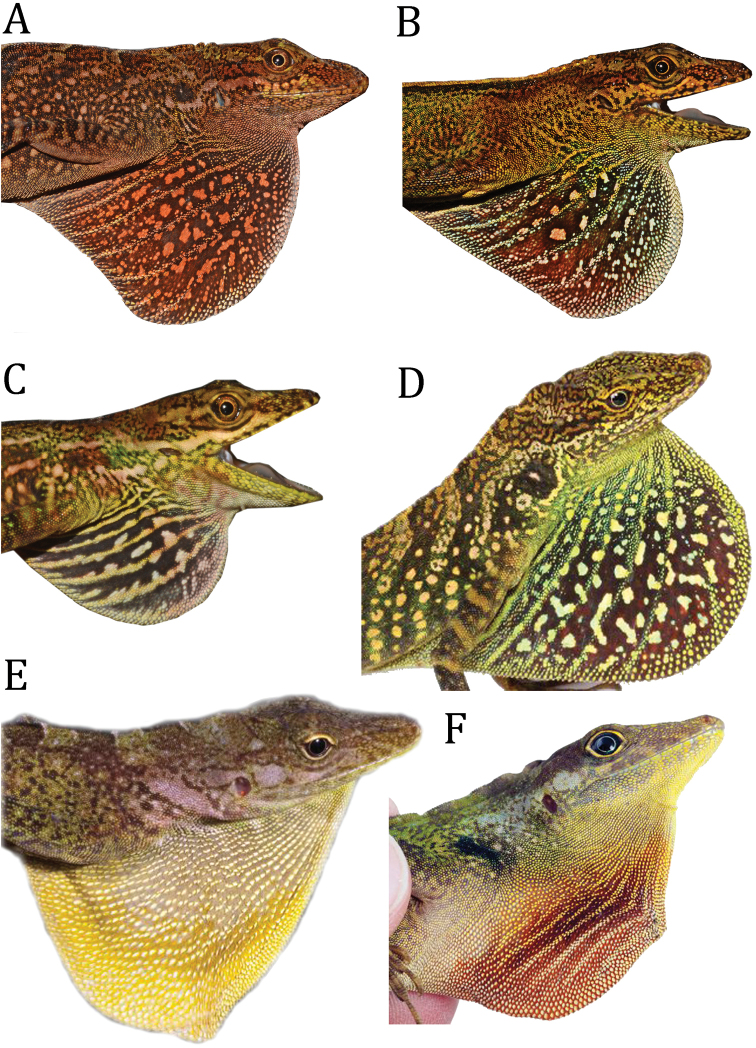
Dewlap of *Anolisdracula* sp. n. and three similar species. **A** male of *A.dracula*, paratype DHMECN 12579, 90.9 mm SVL **B** female of *A.dracula*, paratype DHMECN12587, 80.2 mm SVL **C** subadult female of *A.dracula*, DHMECN 12584, 53.4 mm SVL **D** male of *A.aequatorialis*, QCAZ11605, **E** male of *A.fitchi*, QCAZ8770, 90.5 mm SVL **F** male of *A.podocarpus*, QCAZ10126, 87.0 mm SVL Photographs by Mario Yánez-Muñoz (**A, B, C**), Omar Torres-Carvajal (**D**), Luis Coloma (**E**), and Santiago Ron (**F**).

**Figure 5.**
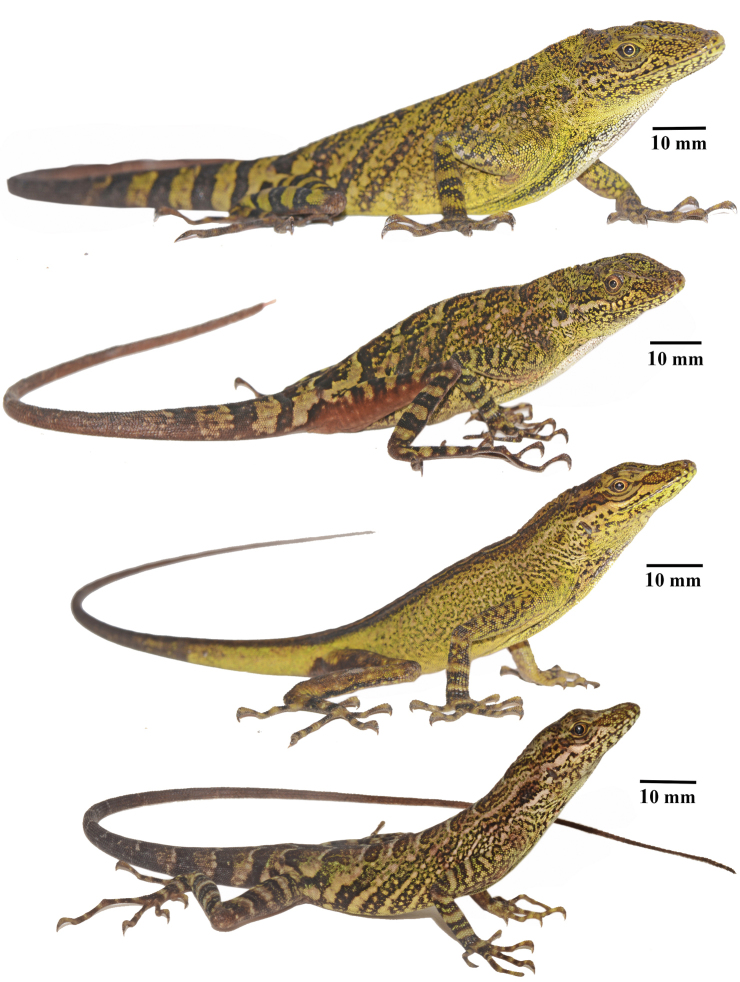
Color variation in *Anolisdracula* sp. n. From top to bottom: Holotype, male DHMECN 12579; male paratype DHMECN 12580; female paratype DHMECN 12760, 72.3 mm SVL; subadult male DHMECN 12578, 70.4 mm SVL. Photographs by Mario Yánez-Muñoz.

**Figure 6.**
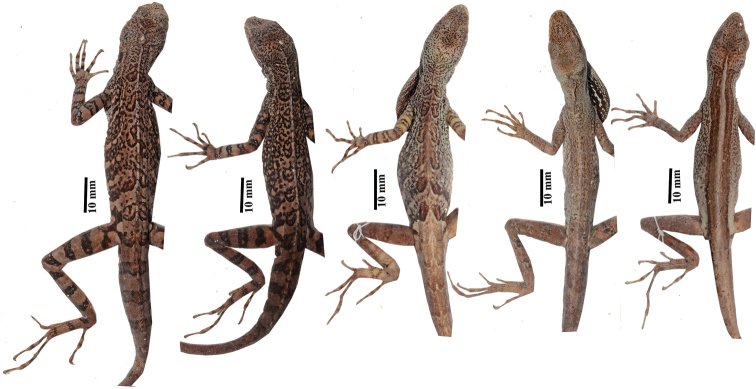
Dorsal color pattern in preservative of *Anolisdracula* sp. n. From left to right: Male paratype DHMECN 12581, 81.6 mm SVL; male paratype DHMECN 12580, 82.8 mm SVL; male paratype DHMECN 12578, 70.4 mm SVL; female paratype DHMECN 12752, 79.6 mm SVL; female paratype DHMECN 12587, 80.2 mm SVL. Photographs by Mario Yánez-Muñoz.

**Figure 7.**
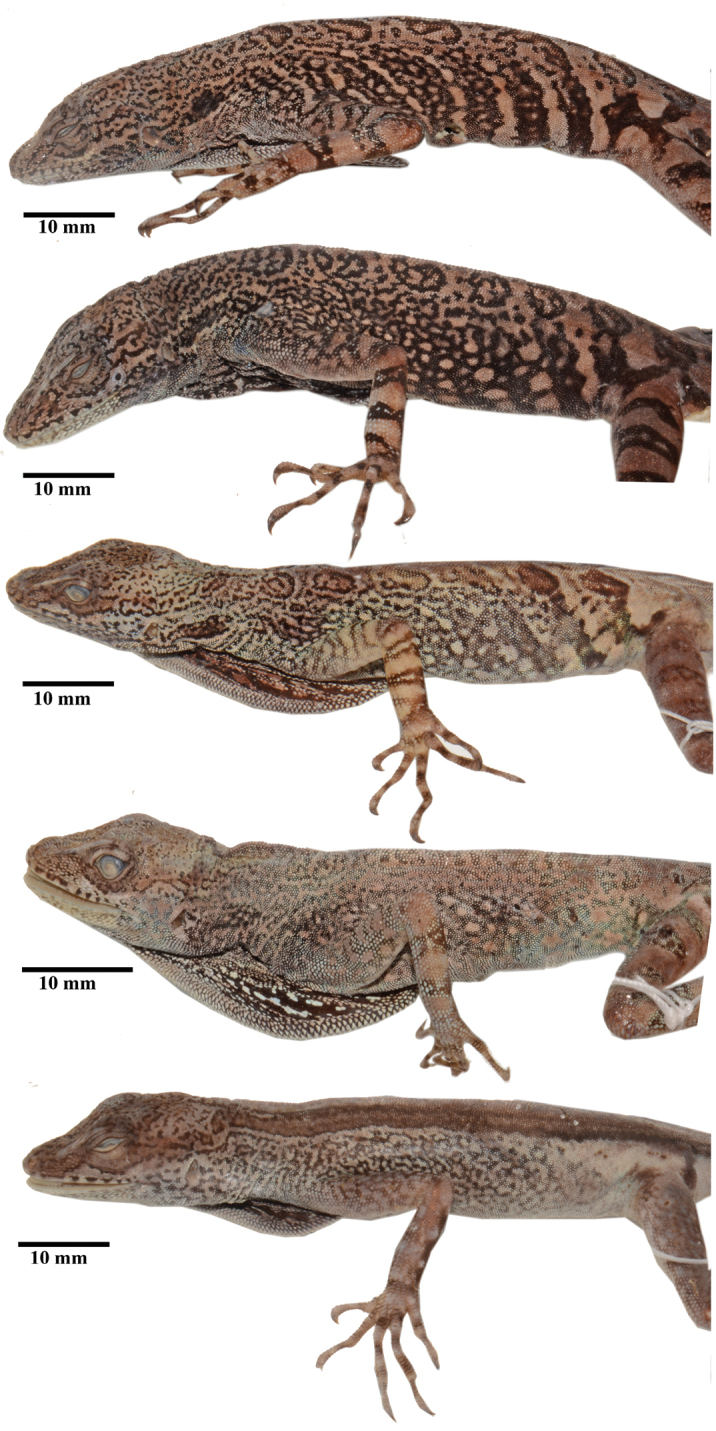
Flank color pattern in preservative of *Anolisdracula* sp. n. From top to bottom: Male paratype DHMECN 12581, 81.6 mm SVL; male paratype DHMECN 12580, 82.8 mm SVL; male paratype DHMECN 12578, 70.4 mm SVL; female paratype DHMECN 12752, 79.6 mm SVL; female paratype DHMECN 12587, 80.2 mm SVL. Photographs by Mario Yánez-Muñoz.

Figs 1 , 2 , 3 , 4 , 5 , 6 , 7 Proposed standard English name: Dracula Anole Proposed standard Spanish name: Anolis dracula

#### Material.

**Holotype.**DHMECN 12579, adult male, from km 18 road Gualpi-Chical, 0°51'8.26"N, 78°13'52.59"W, 2200 m, near Reserva Dracula, Parroquia El Chical, Cantón Tulcán, Provincia Carchi, Ecuador, collected 22 July 2015 by Mario H. Yánez-Muñoz, Juan P. Reyes-Puig, Jorge Brito M., and Héctor Yela. **Paratypes (34).** COLOMBIA (2): ***Departamento Nariño***: Municipio Tumaco: km 6 road Altaquer-Pasto, 1°14'23.44"N, 78°01'09.89"W, 1400 m, Finca de Arcecio, collected 18 August 1991 by Victor Serrano, UVC 10802 (adult male); Reserva Natural Río Nambí, 1°18'0.74"N, 78°04'31.26"W, 1450 m, collected 7 April 2006 by William Beltrán, UVC 16001 (adult male); ECUADOR (32): ***Provincia Carchi*** (30): Cantón Tulcán (30): km 18 road Gualpi-Chical, near Reserva Dracula, Parroquia Chical, 0°51'8.26"N, 78°13'52.59"W, 2200 m, collected 22 July 2015 by Mario H. Yánez-Muñoz, Juan P. Reyes-Puig, Jorge Brito M., and Héctor Yela, DHMECN 12580–81 (adult males), 12583 (subadult male), 12582 and 12584 (juveniles), DHMECN 12760 (female) (same data as holotype); base of Cerro Oscuro, Parroquia El Chical, 0°54'16.43"N, 78°11'29.90"W, 1600 m, collected 21–29 July 2015 by Mario Yánez-Muñoz, Juan Reyes-Puig, Jorge Brito M., and Héctor Yela, DHMECN 12751–53, 12755, 12558 (adult males),12578 (subadult male), 12570, 12756–57, 12759, 12762, 12770 (adult females); Cerro Oscuro, Parroquia El Chical, 0°54'36.07"N, 78°11'4.60"W, 1730 m, collected 23 July 2015 by Mario Yánez-Muñoz, Juan Reyes-Puig, Jorge Brito M., and Héctor Yela, DHMECN 12586–87 (adult males); stream of San José de Río Blanco, 4 km SW El Chical, 0°54’N 78°12’W, 1650 m, collected 16 August 1988 by Doug Wechsler, DHMECN 0369 (adult female); Río San Pablo, near El Chical, Parroquia Chical, 0°54'10.87"N, 78°9'46.22"W, 1399 m, collected 3 July 2011 by Fernando Ayala, Alejandro Arteaga, Lucas Bustamante, Francy Mora, and Paulina Romero, QCAZ 4381, 4384, 4387 (adult females); Sendero Ecológico Teldibi, Parroquia Maldonado, 0°54'46.83"N, 78°6'28.15"W, 1477 m, collected 5 July 2011 by Fernando Ayala, Alejandro Arteaga, Lucas Bustamante, Francy Mora, and Paulina Romero, QCAZ 4405 (subadult male); Sendero Ecológico Teldibi, parroquia Maldonado, 0°54'48.13"N, 78°6'38.26"W, 1389 m, collected 5 July 2011 by Fernando Ayala, Alejandro Arteaga, Lucas Bustamante, Francy Mora, and Paulina Romero, QCAZ 4411 (adult female); Esperanza, Río Pailón, Parroquia El Chical, 0°57'10.69"N, 78°14'18.09"W, 1608 m, collected 27 August 2016 by Diego Almeida, QCAZ 14869–70, 14875–77, 14881 (adult females), QCAZ 14879 (adult male); ***Provincia Imbabura*** (2): Cantón Ibarra (2): Santa Cecilia, Parroquia Lita, 0°50'39.51"N, 78°27'26.64"W, 1600 m, collected 29 July 2017 by Jorge Valencia, FHGO 11282 (adult female), FHGO 10817 (adult male).

**Table 3.** Comparison of lepidotic characters, with Wilcoxon-Mann Whitney tests, between *Anolisdracula* sp. n. and *A.aequatorialis*, from Ecuador and Colombia. For each character the *Z* and *p* values are given, after range and sample size (in parenthesis), and mean/median ± standard deviation for each species. Asterisks indicate the degree of significance, * α = p < 0.05, ** α = p < 0.01.

Character	*A.dracula* sp. n.	* A. aequatorialis *	*Z*	*p*
**Scales between second canthals**	13–17 (34) 14/14.8 ± 1.1	12–17 (31) 16/14.8 ± 0.9	0.89324	0.2153
**Postrostrals**	6–9 (34) 6/6.7 ± 0.9	5–8 (31) 7/6.6 ± 0.9	-0.30575	0.7598
**Loreal rows**	8–11 (34) 9/9.5 ± 0.6	7–10 (31) 9/8.5 ± 0.1	-4.312	1.618E-05***
**Scales between supraorbital semicircles**	2–5 (34) 4/4 ± 0.7	3–5 (31) 4/4.2 ± 0.5	1.3692	0.1709
**Scales between interparietal and semicircles**	5–7 (34) 6/6.1 ± 0.9	5–8 (31) 6/6.6 ± 0.8	2.3944	0.01639*
**Supralabials to below center of eye**	6–8 (34) 7/6.8 ± 0.5	5–7 (31) 6/6.1 ± 0.5	-5.8868	2.19E-08***
**Postmentals**	6–9 (34) 6/6.5 ± 0.9	5–8 (31) 7/6.7 ± 0.7	0.90126	0.3675
**Lamellae under phalanges III–IV of fourth toe**	18–23 (34) 20/20.1 ±1.2	19–23 (31) 21/20.7 ±1.1	2.5284	0.01107*

**Table 4.** Comparison of morphometric characters, with t-test, between *Anolisdracula* sp. n. and *A.aequatorialis*, from Ecuador and Colombia. For each character, the *F*, *t*, and *p* values are given, after range, sample size (in parenthesis), and mean ± standard deviation for each species. Asterisks indicate the degree of significance, * α = p < 0.05, ** α = p < 0.01.

Character	*A.dracula* sp. n.	* A. aequatorialis *	*F*	*p*	*t*	*p*
Head length	16.8–25.7 (34) 20.6 ± 2.2	18.1–25 (31) 21.5 ± 1.9	0.98311	0.9644	2.1842	0.03328*
Head width	8.4–13.1 (34) 11.1 ± 1.2	0.3–15.9 (31) 12 ± 1.2	0.712	0.3549	2.9933	0.003981**
Head height	7.2–16 (34) 10.8 ± 1.6	8.8–14.5 (31) 10.9 ± 1.3	0.6615	0.2609	0.49648	0.6213
Jaw length	12–22.2 (34) 16 ± 2.3	12.3–20.6 (31) 16 ± 1.9	0.73959	0.4111	1.0628	0.2921
Snout length	7.6–13 (34) 9.7 ± 1.2	8.3–12.9 (31) 10.2 ± 1.1	0.71868	0.3682	1.9703	0.05*
Forelimb length	31.2–51.8 (34) 41.3 ± 4.3	35.1–58.8 (31) 45.6 ± 5.4	1.2386	0.5567	3.4396	0.001056**
Hindlimb length	57.4–92.5 (34) 73 ± 7.7	61.9–99.9 (31) 79.1 ± 8.3	0.99276	0.9857	2.999	0.003917**
Axilla-groin length	23.2–45.2 (34) 34.7 ± 4.8	27.5–43.8 (31) 36 ± 4.3	0.66547	0.2677	1.1778	0.2435
Snout-vent length	53.4–91 (34) 76.2 ± 8.5	61.4–106 (31) 82.9 ± 9.2	1.1642	0.6758	2.9648	0.00431**
Tail length	147–256 (34) 201.7 ± 28.6	110–270 (31) 178.3 ± 41.6	0.55363	0.2125	-1.5182	0.1341
Dewlap length	21.5–56 (34) 39 ± 8.4	22.2–59 (31) 40.5 ± 7.3	0.66178	0.2613	0.88446	0.3799
Dewlap height	9.5–31.3 (34) 19 ± 5.9	7.11–39.5 (31) 20.3± 3.3	1.0045	0.9887	0.84217	0.403
Interparietal scale length	1.1–2.2 (34) 1.5 ± 0.3	0.8–1.6 (31) 1.2 ± 0.2	0.98311	0.9644	-4.439	3.8e-05***
Tympanum length	2.1–3.5 (34) 2.6 ± 0.3	2.0–4.1 (31) 2.8 ± 0.4	1.7784	0.1163	2.2627	0.0272*

**Table 5.** Sexual variation in lepidosis and measurements (mm) in *Anolisdracula* sp. n.; range followed by mean and standard deviation.

Character	Males	Females
	*N* = 21	*N* = 13
Scales between second canthals	14–16, 14.7 ± 1	13–17, 14.8 ± 1.2
Postrostrals	6–9, 6.9 ± 1.2	6–8, 6.5 ± 0.7
Row of loreals	9–11, 9.6 ± 0.6	9–11, 9.5 ± 0.6
Scales between supraorbital semicircles	4–5, 4.2 ± 0.4	2–5, 3.7 ± 0.8
Scales between interparietal and semicircles	5–7, 5.7 ± 0.8	5–7, 6.4 ± 0.8
Supralabials to below center of eye	6–8, 6.7 ± 0.6	6–8, 6.8 ± 0.5
Postmentals	6–9, 6.9 ± 1.2	6–7, 6.2 ± 0.4
Lamellae under phalanges III–IV of fourth toe	18–22, 19.6 ± 1.2	19–23, 20.5 ± 1.1
Head length	19.1–25.74, 22 ± 1.9	16.5–21.72, 19.1 ± 1.9
Head width	9–13.1, 11.11 ± 1.3	8.4–11.1, 10.2 ± 0.8
Head height	9.1–16, 12 ± 1.9	7.2–11.2, 9.8 ± 1.1
Jaw length	12–17, 14.2 ± 1.6	14.8–22.2, 17.8 ± 2.8
Snout length	8.6–13, 10.4 ± 1.3	7.6–10.8, 9 ± 1
Forelimb length	39.1–51.8, 46.4 ± 4.1	31.2–44.7, 38.1 ± 3.8
Hindlimb length	57.4–77.5, 67.8 ± 6.3	62.1–92.5, 76.9 ± 8.5
Axilla-groin length	23.2–37.6, 30.6 ± 4.6	30.6–45.2, 36.5 ± 4.5
Snout-vent length	66.6–90.9, 80.2 ± 7.3	53.4–80.2, 69.4 ± 8.6
Tail length	183–256, 213 ± 26.1	147–241, 187.6 ± 30.9

#### Diagnosis.

We assign *Anolisdracula* to the *Dactyloa* clade within *Anolis* (Poe 2004, Poe et al. 2017) based on the following combination of characters: sexual size dimorphism; large body with high numbers of lamellae; more than 20 scales across the snout; Alpha type caudal vertebrae; prefrontal bone separated from nasal; lengthened dentary and loss of angular.

**Figure 8.**
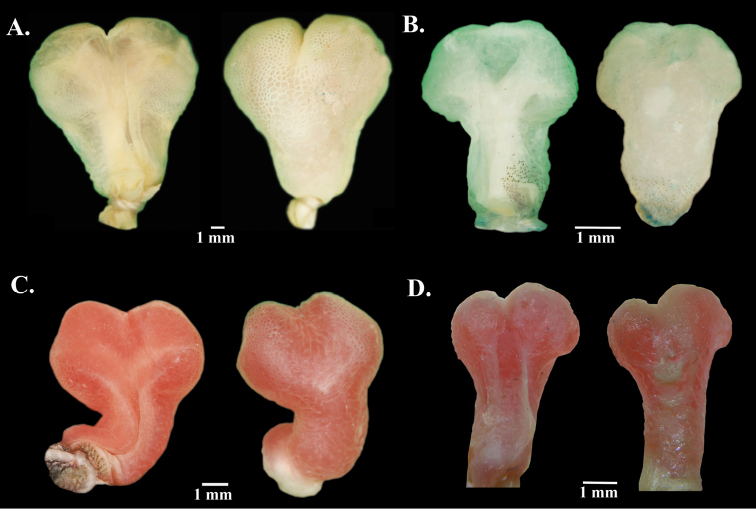
Hemipenes of four species of *Anolis* from Ecuador in sulcate (left) and asulcate (right) views. **A***A.dracula* sp. n. QCAZ 4395, 84.4 mm SVL **B***A.aequatorialis* DHMECN1509, 97.0 mm SVL **C***A.fitchi*DHMECN 5114, 80.0 mm SVL **D***A.podocarpus* QCAZ6038, 90.5 mm SVL.

**Figure 9.**
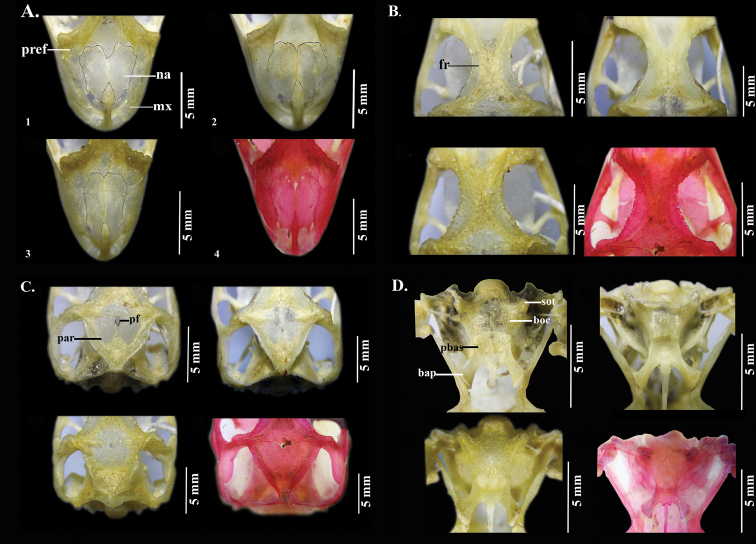
Cranial osteology of **1***Anolisdracula* sp. n. (DHMECN 12586) **2***A.aequatorialis* (DHMECN 7623) **3***A.fitchi* (DHMECN 9247) **4***A.podocarpus* (QCAZR 6047). **A** dorsal view of snout region; **B** dorsal view of frontal region **C** dorsal view of parietal region **D** ventral view of occipital region. Abbreviations: bap, basipterygoid process; boc, basioccipital; pf, pineal foramen; fr, frontal; mx, maxillae; na, nasals; par, parietal; pbas, parabasisphenoid; pref, prefrontal; sot, sphenoccipital tubercle. Photographs by Carolina Reyes-Puig.

**Figure 10.**
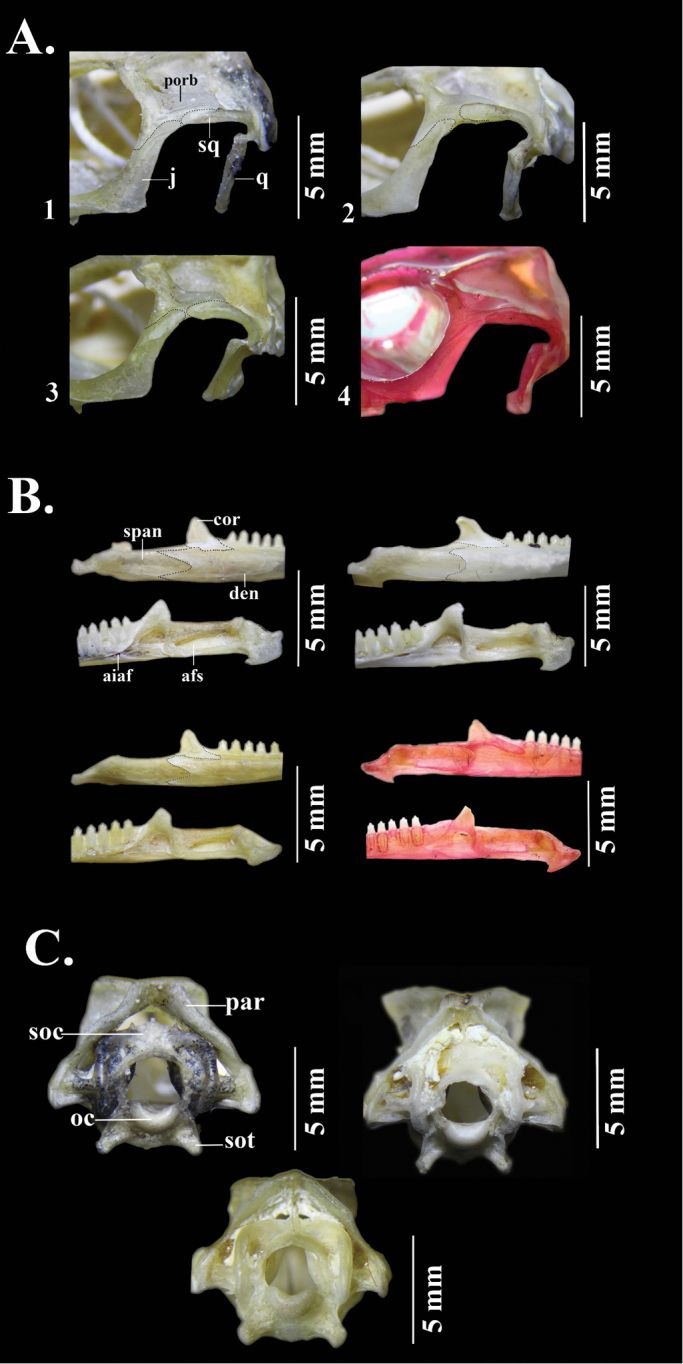
Cranial osteology of **1***Anolisdracula* sp. n. (DHMECN 12586) **2***A.aequatorialis* (DHMECN 7623) **3***A.fitchi* (DHMECN 9247) **4***A.podocarpus* (QCAZR 6047) **A** lateral view of postorbital region **B** lateral (top) and medial (bottom) views of the mandible **C** posterior view of cranium. Abbreviations: afs, adductor fossa; aiaf, anterior inferior alveolar foramen; cor, coronoid; den, dentary; j, jugal; oc, occipital condyle; par, parietal; porb, postorbital; q, quadrate; soc, supraoccipital; sot, sphenoccipital tubercle; span, supra-angular; sq, squamosal. Photographs by Carolina Reyes-Puig.

**Figure 11.**
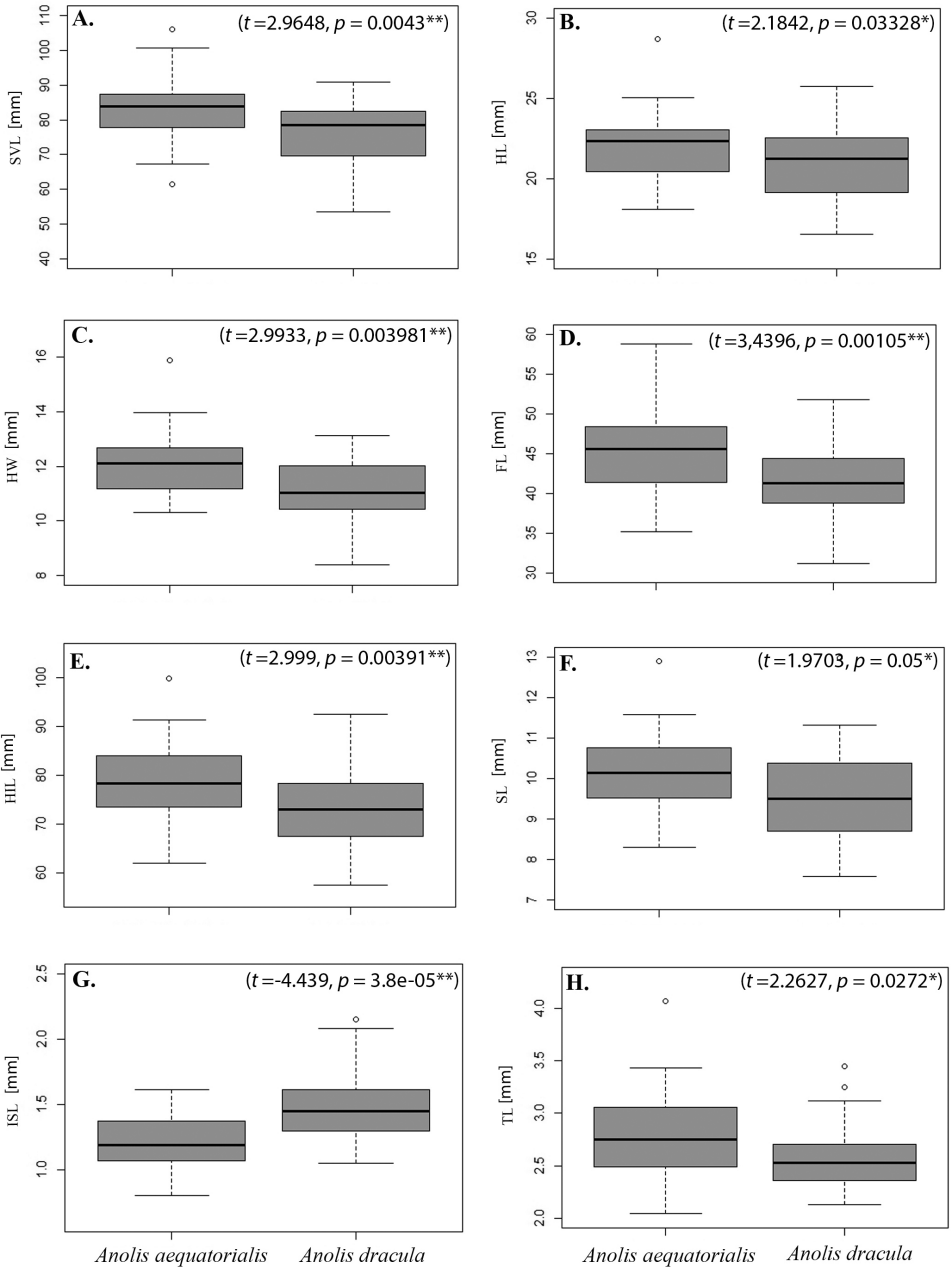
Box plots of eight variables in *Anolisdracula* sp. n. and *A.aequatorialis*. **A** Snout-vent length **B** Head length **C** Head width **D** Forelimb length **E** Hindlimb length **F** Snout length **G** Interparietal scale length **H** Tympanum length.

*Anolisdracula* is most similar in morphology and coloration to *A.aequatorialis* (character states in parentheses), but differs from it in the following characters: large and robust hemipenes, 14 mm (4.7 mm; *W* = 0; *p* = 0.004), with a well-developed spermatic sulcus (hemipenis small; Figure 8); well-developed parietal crests, bowed outwards and projected laterally (relatively straight parietal crests, without laterally extending edges) (Figure 9); pineal foramen large, oval (rounded and small), and contacting fronto-parietal fissure (pineal foramen not contacting fronto-parietal fissure; Figure 9); rugose (smooth) basioccipital and sphenoccipital tubercles; jugal and squamosal in contact (separated by postorbital; Figure 10); posterior edge of dentary extending over more than a quarter of supra-angular (1/8 the size of supra-angular; Figure 10); dewlap scales cream (green or yellowish green) and in seven (10) rows in males, yellow or turquoise (green or yellowish green) and in five (six) rows in females (Figure 4); edge of dewlap cream (green or yellowish green); dewlap background brown or reddish brown (yellowish green to black), with orange (yellowish green, turquoise or yellowish orange) spots in males; dewlap background reddish brown to black (dark brown to black) in females; throat and chin cream splashed with dark brown (yellowish green); some males exhibit a lateral dark brown ocellus on neck, similar in size to eye (green, turquoise or brown, larger than eye); some females bear a dorsal, longitudinal brown stripe (absent; Figure 3); dark transverse bands on limbs of females weakly defined or absent (limb bands well defined in females, Figs 5, 6); smaller body size, 76.2 ± 8.5 mm SVL, (82.9 ± 9.2 mm; *t* = 2.96; *p* = 0.00431); shorter head, 20.6 ± 2.2 mm head length (21.5 ± 1.9; *t* = 2.18; *p* = 0.03328); narrower head, 11.1 ± 1.2 mm head width (12.0 ± 1.2 mm; *t* = 2.99; *p* = 0.004); shorter forelimbs, 41.4±4.3 mm (45.6 ± 5.4 mm; *t* = 3.44; *p* = 0.001); shorter hind limbs, 73.0 ± 7.7 mm (79.1 ± 8.3 mm; *t* = 2.999; *p* = 0.004); larger interparietal scale, 1.48 ± 0.25 mm in length (1.22 ± 0.2 mm; *t* = -4.439; *p* = -3.85 e-05); narrower tympanum, 2.6 ± 0.3 mm in length (2.8 ± 0.4 mm; *t* = 2.29; *p* = 0.027) (Figure 11; Tables 3–4).

Among dactyloid species from Ecuador and Colombia, *Anolisdracula* is similar in color and morphology to *A.fitchi* and *A.podocarpus*. However, both species occur east of the Andes in Ecuador and they can be distinguished (character states in parentheses) from *A.dracula* by the following characters: hemipenis with slightly defined lobules, which means that the outline of the lobules are not clearly distinguishable from the trunk (lobules well defined), and twice as long as hemipenes of *A.fitchi* and *A.podocarpus*, hemipenis length in *A.dracula* 14 mm (*A.fitchi* 7 mm; *A.podocarpus* 6 mm; Figure 8); well-developed parietal crests, bowed outwards and projected laterally (irregular, with curved edges in *A.fitchi*; relatively straight in *A.podocarpus*; Figure 9); large and oval pineal foramen (small and rounded in *A.fitchi* and *A.podocarpus*); smooth lateral edges of frontal bone (serrated in *A.fitchi* and *A.podocarpus*; Figure 9); short nasal bones (elongated in *A.fitchi* and *A.podocarpus*; Figure 9); lateral projections on posterolateral edges of parietal crests (no lateral projections in *A.fitchi*); strongly rugose surface of basioccipital and sphenoccipital tubercles (rugose in *A.fitchi* and slightly rugose in *A.podocarpus*); jugal and squamosal bones in contact (separated by postorbital bone in *A.fitchi* and *A.podocarpus*; Figure 10); posterior edge of dentary extending ¼ length of suprangular (same in *A.fitchi* and ⅛ of suprangular length in *A.podocarpus*); a poorly developed nuchal crest in males (well defined in *A.fitchi* and *A.podocarpus*); brown iris with a golden ring (turquoise blue with a white ring in *A.podocarpus*, bluish grey with golden ring in *A.fitchi*); large interspaces of naked skin among dewlap scale rows (reduced interspaces in *A.fitchi* and *A.podocarpus*; Figure 4); uniformly brown or reddish brown dewlap with cream edges and spots varying from turquoise to light brown in females and orange spots in males (*A.fitchi* with yellowish-brown dewlap, with dark brown edges and throat, and in *A.podocarpus* reddish-brown dewlap with dark brown anteriorly and pink posteriorly; Figure 4).

Other *Dactyloa* species distributed in the lowlands and foothills of western Ecuador and Colombia and somewhat similar to *Anolisdracula* are *A.chloris*, *A.fasciatus*, *A.gemmosus*, *A.otongae*, *A.parilis*, *A.poei* and *A.ventrimaculatus*. However, these species are smaller in SVL (range between 56 – 80 mm) and hemipenial length than *A.dracula* and have dewlaps with a white background (brown or reddish brown in *A.dracula*).

Finally, although the average ND2 genetic distance between *A.dracula* and its closest relative *A.aequatorialis* is relatively low (0.049), it is comparable to DNA divergences between other species pairs, such as *Anolisheterodermus* versus *Anolisinderenae* (0.042) and *Anolisanatoloros* versus *Anolisjacare* (0.041).

#### Description of holotype

**(paratype data in parentheses).***Head*: Frontal depression present; head dorsal scales small and keeled in frontal and nasal regions; internasals smooth; parietal region with granular scales; post-rostrals seven (6–9), fourth enlarged; nasal contacting rostral; circumnasal round, separated from rostral by one scale; external naris separated from rostral by three scales, not contacting supralabial; supraorbitals larger than adjacent scales, polygonal, rugose, and separated by two scales from supraorbital semicircles; supraocular disk with small, keeled scales of similar size; parietals heterogeneous in size, slightly quadrangular and keeled; scales between interparietal and supraorbital semicircles heterogeneous in size; interparietal larger than wide, slightly rhomboid, much larger than adjacent scales (10 ×), similar in size to ear opening, and separated by 2–3 small scales from supraorbital semicircles; scales between interparietal and nape 13; parietal scales keeled; canthals keeled; nasal scale single; canthal scales nine (8–9); anterior canthals contacting circumnasals; scales between first canthals 17 (14–17); scales between second canthals 14 (13–17); loreal rows 8 (8–11), keeled, horizontal, upper contacting canthals; preoculars four; subocular scales seven, separated from supralabials by 1–2 scale rows; temporals small and granular, not in rows or series; supralabials seven (6–8); ear opening oval-shaped, surrounded by small granular scales; anterior edge of rostral ventrally visible; mental semicircular, concave and divided; infralabials in seven rows; sublabials absent; postmentals 9 (6–9).

*Dewlap*: 56 mm long and 31.3 mm high (males 46 ± 7 mm [33–56] in length, 22.8 ± 4.8 mm [17.3–30.4] in width, n = 21; females 34 ± 8 mm [21.5–49] in length, 15 ± 4 mm [10–23] in width, n = 13); dewlap extending posterior to arms in males and slightly beyond the insertion of the arms in females; dewlap longitudinal scale rows seven (5–8), separated by naked skin; clusters of dewlap scales broad and colored.

*Trunk*: Middorsal and paravertebral scales small and keeled, slightly larger than flanking scales, which are granular/conical and separated by small skin interspaces; ventral scales smooth, subimbricate, larger than dorsals; groin, axilla and neck covered by granular scales; nuchal and dorsal folds present, reduced in females; two enlarged postanals in males.

*Limbs*: Fore and hind limbs with keeled scales; hind limbs more robust, 1.8 times longer than forelimbs; lamellae of subdigital pad of fourth toe 19 (18–23; counted in the manner of Williams et al. 1995).

*Tail*: Cylindrical, with keeled scales at the base, others imbricated; 125% longer than snout-vent length.

*Color in life* (holotype and paratypes): *Anolisdracula* is chromatically variable depending on sex, emotional stress, and perch type (Figure 5). Males dorsum with dark brown transverse bands delineated by black and separated by greenish brown, or light brown or black bands separated by cream (Figs 6, 9); females dorsum varies, from light green with dark green V-shaped transverse bands separated by pinkish cream, turquoise cream or whitish cream lines (Figure 5), to beige or dark brown with darker brown transverse bands separated by whitish coloration (Figs 5, 6); all morphs exhibit a light brown or black hourglass-shaped spot on insertion of forelimbs; tail dark green with bands separated by pink spaces in females and light green or dark brown in males; belly usually cream; throat cream with light green small spots in females and immaculate in males; iris copper; tongue cream; in males naked skin of gular sac dark brown, with bright turquoise to bright green scattered irregular markings, longitudinal rows of sac scales green; in females, naked skin of gular sac brown, with irregular bright turquoise to bright brown scattered markings, longitudinal rows of sac scales turquoise.

*Color in preservative* (holotype and paratypes preserved between two and ten years): Dorsum in males bluish grey, flanks whitish pale-blue, with light or black hourglass-shaped spots, belly grey or bluish cream (Figs 6–7); dorsum in females bluish grey, separated by light bluish-cream transverse bands on flanks, with black or white spots, belly cream; both sexes with black visceral peritoneum.

*Hemipenis* (Figure 8): Hemipenis bilobed, 14 mm in length; trunk becoming distinctly wider distally; lobules short and rounded; asulcate side with a semicircular constriction in first quarter of trunk; sulcus spermaticus wide, with thick fringes, branching at base of lobules and extending to base of transversal veil on sulcate side of lobules; apical and asulcate surfaces of lobules covered by calyces; asulcate region of trunk and proximal region of lobules with thin transverse folds; surface of constriction separating stem from apex with small calyces and folds.

*Skull* (based on DHMECN 12586; Figs 9–10): Cephalic casque absent; parietal roof flat and slightly convex, with a depression in postparietal region, the crests meet posteriorly and are bowed outwards, projected laterally, with crenulations on edges, and anterolateral corners extending with posterolateral edges of frontal; pineal foramen contacting fronto-parietal suture; postfrontal present; frontal bones rugose, with blunt supraorbital edges; prefrontal contacting nasal between frontal and maxilla; no parallel crests on nasals; nasal bones convex, slender, and elevated in middorsal region; external nares bordered posteriorly by nasals; nasals slightly overlapping premaxilla; jugal and squamosal in contact; posterodorsal ramus of squamosal moderately long; sphenoccipital tubercles slightly raised; basipterygoid process short and wide; occipital, basioccipital and parabasisphenoid wider than long and rugose; splenial present; process of coronoid extending posteriorly; external opening of surangular foramen entirely within surangular; posterior suture of dentary extending beyond posterior edge of coronoid process; angular process of dentary present; skull longer and higher than wide.

#### Distribution and natural history.

*Anolisdracula* occurs on the foothills of the Andes of southwestern of Colombia and northwestern Ecuador. It has been recorded in the provinces of Carchi and Imbabura, Ecuador, and the department of Nariño, Colombia, between 1187–2353 m in elevation. The known distributional area of *A.dracula* is relatively small, approximately 1582 km^2^ (Figure 13), and all records are from evergreen low montane forest (Cuesta et al. 2009, MAE 2013).

*Anolisdracula* was the most common species of anole during surveys conducted by the Herpetological Division from Instituto Nacional de Biodiversidad de Ecuador during the June-August period in 2015 and 2016 at Cerro Oscuro in the Dracula Reserve. Specimens were collected in mature and secondary forests, degraded areas with pastures and native vegetation, as well as along the edges of secondary roads. Almost all specimens were found sleeping at night on leaves of Araceae, Arecaceae, and pteridophytes, between 0.6 and 2.3 m above the ground.

Occasional observations during 2016 (March-June) suggest that *A.dracula* shows sleeping-perch fidelity and is active on the ground. A female was observed sleeping on the same leaf of Araceae for two consecutive nights. In the same field trip, we observed two females in clear and sunny days starting thermoregulatory behavior at 7 am, with slight head movements and small jumps between branches. As the sun rose, the females moved down to the ground. A male was observed foraging on leaf litter at noon. In addition, several specimens were collected in pitfall traps. Some individuals were observed on leaf litter during the day, with cryptic coloration (brown color), whereas at night, most specimens were greenish.

Stomach contents revealed at least 10 prey items and three species of parasites. The most diverse prey was Coleoptera (4 spp.), followed by Hymenoptera (3 spp., including two Formicidae), Arachnida (1 sp.), Diptera (1 sp.), and Lepidoptera (1 sp.). Ants (Hymenoptera) were the most frequent stomach content (44%), followed by Nematoda (21%), plant material (14%), and Coleoptera, Opiliones, Araneidae, and Diptera (21%).

#### Etymology.

The specific epithet *dracula* it is a noun in apposition that refers to the Dracula Reserve, located within the distribution of the new species and near its type locality. The Dracula Reserve is an initiative of the EcoMinga Foundation, sponsored by the Orchid Conservation Alliance, Rainforest Trust, University of Basel Botanical Garden, and their individual donors. The Reserve protects an area with a high diversity of orchids of the genus *Dracula*.

#### Phylogenetic relationships.

**Figure 12.**
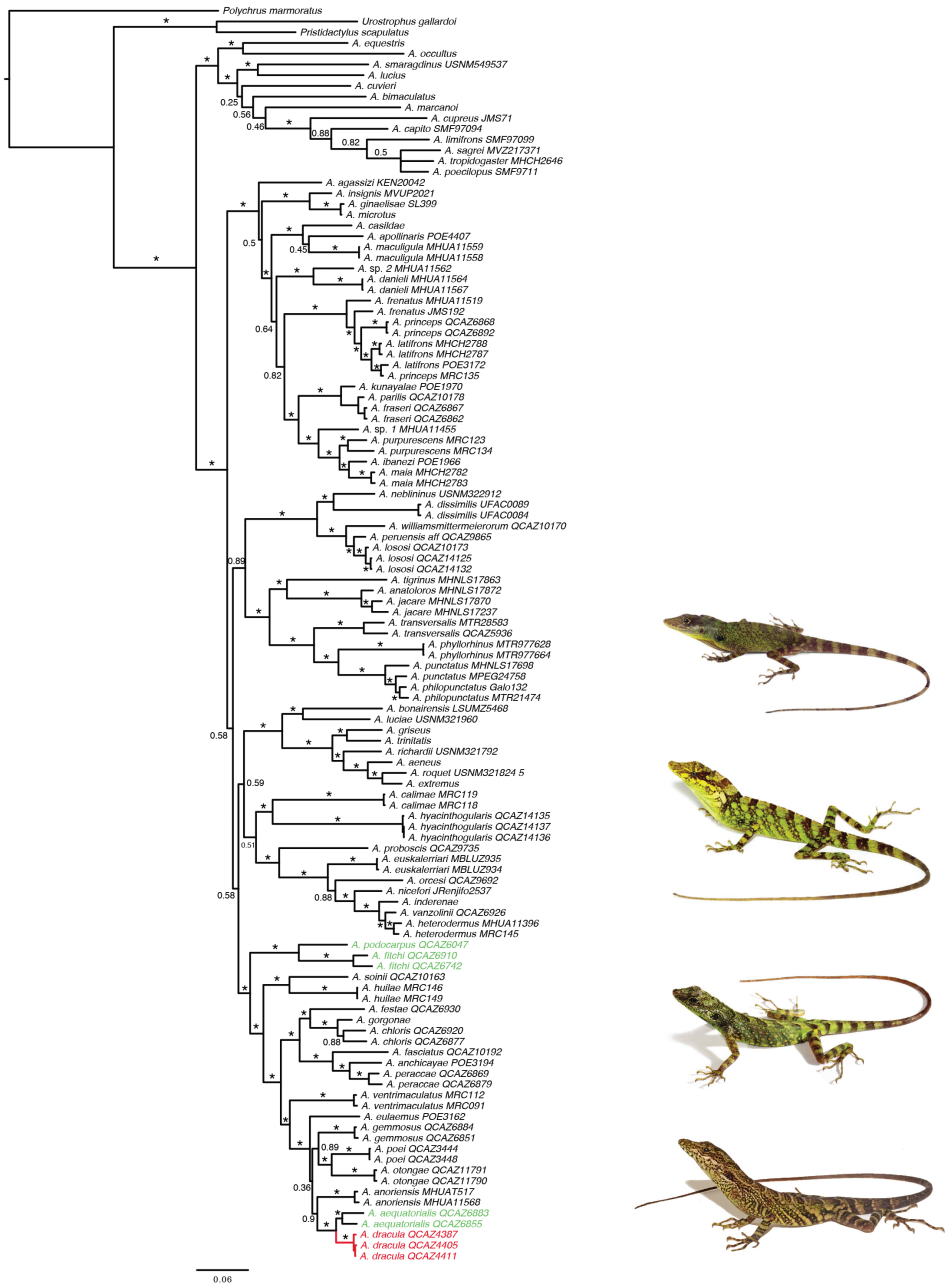
Phylogeny of *Dactyloa* including *Anolisdracula* sp. n., 50% majority-rule consensus tree obtained from a Bayesian analysis of 117 specimens, two mitochondrial genes (COI, ND2) and one nuclear gene (RAG1). Numbers above branches correspond to Bayesian posterior probability (PP) values; asterisks represent PP ≥ 0.95; scale bar corresponds to the mean number of nucleotide substitutions per site. Photographs from top to bottom: *Anolispodocarpus* (Santiago R. Ron-BIOWEB), *A.fitchi* (Juan C. Sánchez-BIOWEB), *A.aequatorialis* (Diego Quirola-BIOWEB), *A.dracula* (Mario Yánez-Muñoz).

The Bayesian analysis estimated *Anolisdracula* to be sister to *A.aequatorialis*, with strong support (Figure 12). This relationship was expected, as these species are very similar in morphology and coloration (Figs 2–4). The above clade is sister to *A.anoriensis*, and within a strongly supported clade also containing *A.gemmosus*, *A.otongae*, *A.poei*, *A.eulaemus*, *A.ventrimaculatus*, *A.peraccae*, *A.anchicayae*, *A.fasciatus*, *A.chloris*, *A.gorgonae*, and *A.festae*, all representing an important radiation of the *Dactyloa* clade of *Anolis* along the western slopes of the Tropical Andes in northwestern South America (Figure 12).

**Figure 13.**
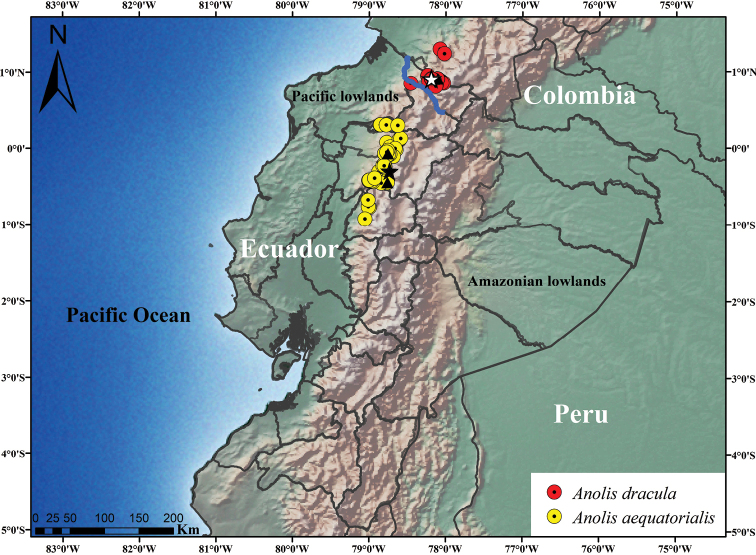
Distribution of *Anolisdracula* sp. n. and *A.aequatorialis*. White star represents the type locality of *A.dracula* and the black star the type locality of *A.aequatorialis*. The blue line corresponds to the Mira River Basin. The black triangles indicate samples used in the phylogeny for each species. The source of the raster layer for the map is from naturalearthdata.com.

## Discussion

Although Ayala-Varela and Velasco (2010) reported *Anolisaequatorialis* from southwestern Colombia, we found that the specimens on which such a distribution was based (UVC 10802, UVC 16001) correspond to specimens of *A.dracula*. Recently, Cisneros-Heredia (2017) determined that the type locality of *A.aequatorialis* is located in the western slopes of the Andes in northern Ecuador, in the province of Pichincha. Based on our sampling and phylogenetic analyses (Figure 12), we infer that typical populations of *A.aequatorialis* are located only in Ecuador.

*Anolisdracula* is nested within the *Dactyloa* clade of *Anolis*, which is in turn well supported by morphological and molecular data (Castañeda and de Queiroz 2013; Prates et al. 2015; Poe et al. 2017). Castañeda and de Queiroz (2013) proposed five species series within the *Dactyloa* clade: *A.aequatorialis* series, *A.latifrons* series, *A.punctatus* series, *A.heterodermus* series, and *A.roquet* series. The species content of each series was updated by Poe et al. (2017, p. 687). Based on its phylogenetic position (Figure 12) and morphology (moderate to large size [73.6−96.0 mm SVL], with narrow toe lamellae), we conclude that *A.dracula* belongs to the *A.aequatorialis* series (Williams 1976).

Unlike many recently described species of anoles (e.g., Poe et al. 2009; Velasco et al. 2010; Torres-Carvajal et al. 2018) from Ecuador and Colombia, we refer to the new species described herein as cryptic, due to the very similar morphology and color pattern (including dewlap) to *Anolisaequatorialis*. This finding suggests that other cryptic species of anoles from the Tropical Andes are yet to be discovered, as has been the case in other taxa (e.g. Guayasamin et al. 2015; Arteaga et al. 2016; Guayasamin et al. 2017), and we highlight the importance of building modern specimen collections of supposedly known species (e.g., *A.aequatorialis*), particularly from poorly explored areas. After extensive geographical sampling, we conclude that the new species described in this paper occurs only north of the Mira River Basin in northern Ecuador (Figure 13). This basin has been recognized as an important isolation barrier for small vertebrates in western Ecuador (Arteaga et al. 2016), which possibly explains the split between *A.aequatorialis* and *A.dracula* by vicariance and subsequent allopatric speciation, mainly because large rivers can act as geographical barriers that may permit genetic differentiation between populations (Ron 2000).

The hemipenis of *Anolisdracula* is proportionally larger than that of its sister species, *A.aequatorialis*. However, further studies are necessary to evaluate whether this sexual morphological difference may have led to the divergence of these two highly cryptic sister species.

## Supplementary Material

XML Treatment for Anolis dracula

## Appendix I

**Additional specimens examined**

***Anolisdracula* sp. n.: Carchi**: DHMECN 12754, adult male; DHEMCN 12575, juvenile female; DHMECN 12771 juvenile, DHMECN 12567, hatchling, Cerro Oscuro, El Chical, 1600 m; DHMECN 12588–90, Cerro Oscuro, 1730 m. QCAZ 14925–14926, adult females, El Rosal, El Goaltal, 1351 m; QCAZ 14908, 14921, adult females, QCAZ 14923, 14919, adult males, quebrada sector Santa Rosa, 1482 m; QCAZ 14905, 14916, adult males, quebrada sector Santa Rosa, 1433 m; QCAZ; QCAZ 14911, adult male, 14920, adult female, Quebrada N°1, quebrada sector Santa Rosa, 1502 m; QCAZ 14934–14935, adult female, Quebrada N°2 De Piedras, El Goaltal, 1612 m; QCAZ 15219, adult female, Bosque Protector Cerro Golondrinas, 2264 m; QCAZ 15211, adult male, Bosque Protector Cerro Golondrinas, 2197 m; QCAZ 15218, adult female, Bosque Protector Cerro Golondrinas, 2187 m; QCAZ 15196, adult female, Bosque Protector Cerro Golondrinas, 2335 m; QCAZ 15213, adult male, Bosque Protector Cerro Golondrinas, 2182 m; QCAZ 15220, adult male, Bosque Protector Cerro Golondrinas, 2169 m; QCAZ 15214, 15216, adult females, Bosque Protector Cerro Golondrinas, 2150 m; QCAZ 15209, adult female, Bosque Protector Cerro Golondrinas, 2103 m; QCAZ 8711, adult male, near to Chilma Bajo, 2250 m; QCAZ 8707, adult male, near to Chilma Bajo, 2100 m; QCAZ 8699, adult female, near to Chilma Bajo, 2058 m; QCAZ 8665-8668, 8676, adult females, QCAZ 8695, adult male, Finca de Aníbal Pozo, Chilma Bajo, 2071 m; QCAZ 14885, 14887, adult males; QCAZ 14882, adult female, Sendero Río Pablo hacia Cerro Golondrinas,1722 m; QCAZ 11781, adult, QCAZ 11782, adult female, La Centella, 2168 m, 1635 m; QCAZ 14202, adult male, Río Verde y Río Pablo, Río Estrellita (Guapil), 1618 m; QCAZ 4394, adult male, QCAZ 4395, adult, Río San Pablo, near to Chical, 1428 m, July 3, 2011; QCAZ 4022, adult female, 1 km de Maldonado, 1500 m; QCAZ 4403, 4407, 4409, 4412, 4365, adult females, QCAZ 4361, males adult; QCAZ 3701, adult male, Sendero Ecológico Teldibi, 1466 m; QCAZ 12269, adult male, Sendero Ecológico Teldibi, 1505 m; QCAZ 4413, adult female, Sendero Ecológico Teldibi, 1389 m; QCAZ 14873, adult female, Esperanza, Río Pailón, 1648 m; QCAZ 14871, adult female, Esperanza, Río Pailón, 1609 m; QCAZ 15227, adult male, Sector Río Pailón, 1610 m; QCAZ 15228, adult female, Sector Río Pailón, 1610 m.

***Anolisaequatorialis*: Pichincha**: QCAZ 11847, adult male, Las Tolas, 1760 m; QCAZ 7800, adult, Estación Biológica Río Bravo, Mindo, 1556 m, July 27, 2006; QCAZ 6861, adult female, Mindo, en el camino entre Mariposas de Mindo y Mindo Garden, 1360 m; QCAZ 6855, adult male, Mindo, road Mariposas de Mindo y Mindo Garden, 1328 m, January 18, 2006; QCAZ 6863, adult male, Mindo, Mindo Garden, 1191 m; QCAZ 6883, adult male, On the bank of Río Chisinche, on road to Conchacato, 1692 m; QCAZ 6890, adult female, On the bank of Río Chisinche, on road to Conchacato, 1694 m; DHMECN 4156, 5499, adult males, Quito, Nanegallito, Reserva Orquideológica Pahuma; DHMECN 4267, adult male, Nono, Reserva Biológica Tamboquinde; 5762, adult male, Quito, Pacto, Las tolas, 1200–1600 m; DHMECN 5830, 5834, adult males, Quito, Saragoza La Unión Río Cinto; DHMECN 5761, 5764–67, adult females, Quito, Pacto, Las Tolas, 1200–1600m; DHMECN 5831–32, adult females, Quito, Saragoza, La Unión Río Cinto; DHMECN 8818, adult female, Calacalí, El Golán, 1962 m. **Cotopaxi**: DHMECN 1629, adult female, Pangua, Hacienda La Mariela. **Santo Domingo de los Tsáchilas**: QCAZ 2784, adult male, near to Chiriboga, Las Palmeras, La Soledad, Estación Científica Río Guajalito; QCAZ 773, adult male; DHMECN 1508, adult female, Chiriboga Estación Experimental “La Favorita”, 1800 m; DHMECN 7622–7624, adults, Mejía, Reserva Río Guajalito, 1800 m; DHMECN 1509, adult male, Lloa, Chiriboga Estación Experimental “La Favorita”, 1800 m.

***Anolisfitchi*: Tungurahua**: DHMECN 5114, adult male, Reserva Río Zuñag, 1269 m; **Napo**: DHMECN 9247, adult male, Santa Rosa, 1300 m.

***Anolispodocarpus*: Zamora Chinchipe**: QCAZ 6038, 6047, adult, Parque Nacional Podocarpus, Romerillos, 1593 m.
